# Activation Pathways of Murine Macrophages by Lipophosphoglycan from Strains of *Leishmania major* (FV1 and LV39)

**DOI:** 10.1021/acsinfecdis.4c00295

**Published:** 2024-09-23

**Authors:** Vanessa Mançur Santos, Astrid Madeleine Calero Goicochea, Antônio José Soares Neto, Flávio Henrique Jesus Santos, Jéssica Lobo da Silva, Théo Araújo-Santos, Leonardo Paiva Farias, Claudia Ida Brodskyn, Valéria M. Borges, Rodrigo Pedro Soares, Jonilson Berlink Lima

**Affiliations:** † Instituto Gonçalo Moniz, Fundação Oswaldo Cruz (FIOCRUZ), Salvador, BA 40296-710, Brasil; ‡ Faculdade de Medicina, Universidade Federal da Bahia (UFBA), Salvador, BA 40.026-010, Brasil; § Núcleo de Agentes Infecciosos e Vetores (NAIVE), Universidade Federal do Oeste da Bahia (UFOB), Barreiras, BA 47808-021, Brasil; ∥ Instituto René Rachou, Fundação Oswaldo Cruz (FIOCRUZ), Belo Horizonte, MG 30.190-009, Brasil; # Laboratório de Medicina e Saúde Pública de Precisão (MeSP2), Instituto Gonçalo Moniz, Fundação Oswaldo Cruz, Salvador, BA 40296-710, Brazil

**Keywords:** Lipophosphoglycan, macrophage, leishmaniasis, *Leishmania major*, inflammatory mediators, cytokines

## Abstract

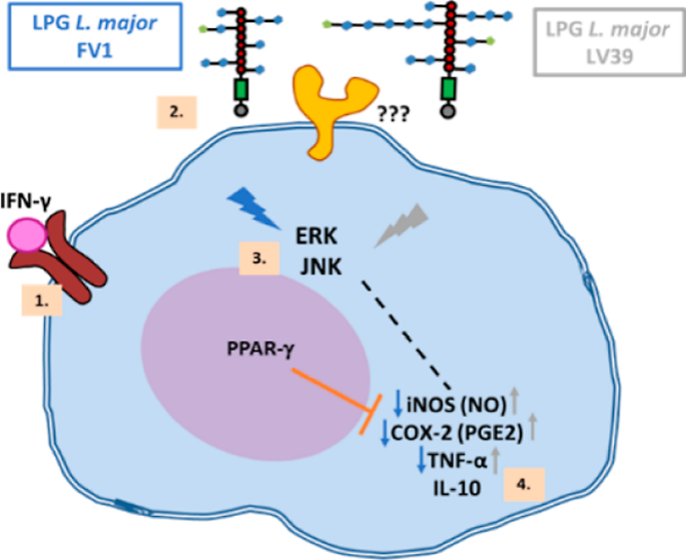

Lipophosphoglycan (LPG) is an important *Leishmania* virulence factor. It is the most abundant surface glycoconjugate in promastigotes, playing an important role in the interaction with phagocytic cells. While LPG is known to modulate the macrophage immune response during infection, the activation mechanisms triggered by this glycoconjugate have not been fully elucidated. This work investigated the role that LPGs purified from two strains of *Leishmania major* (FV1 and LV39) play in macrophage activation, considering the differences in their biochemical structures. Bone marrow-derived macrophages from BALB/c mice were stimulated with 10 μg/mL purified LPG from the LV39 and FV1 strains. We then measured the production of nitric oxide (NO) and cytokines, the expression of inducible nitric oxide synthase (iNOS) and cyclooxygenase-2 (COX-2), and the activation of MAPK pathways. LPG from the LV39 strain, which has longer poly-galactosylated side chains, induced a more pro-inflammatory profile than that from the FV1 strain. This included higher production of NO, TNF-α, and PGE2, and increased expression of COX-2 and iNOS. Additionally, the phosphorylation of ERK-1/2 and JNK was elevated in macrophages exposed to LPG from the LV39 strain. No difference in IL-10 production was observed in cells stimulated by both LPG. Thus, intraspecific structural differences in LPG contribute to distinct innate immune responses in macrophages.

*Leishmania major* is the main species that causes cutaneous leishmaniasis (CL) in the Old World and, more recently, in the New World.^1^ Although some studies have demonstrated differences in the pathogenesis of leishmaniasis caused by various strains of this species, most factors responsible for this phenomenon remain unknown.^2^ This variation in disease outcome may result from interactions between parasite virulence factors (genetic, biochemical) and vertebrate host factors (such as race, age, and nutritional and immune responses).^3−5^ Together, these features can determine the persistence or cure of the disease.

As the main host cells for *Leishmania* spp., macrophages play a fundamental role in the immunopathogenesis of the disease. These cells activate inflammatory defense mechanisms, such as the production of reactive oxygen and nitrogen species, the production of IL-1β, and the modulation of lipid metabolism.^6,7^ However, parasites and their constituents can modulate signaling pathways as a survival mechanism.^8,9^ Numerous studies involving *L. major* have provided insights into the polarization of T helper 1 (Th1) and T helper 2 (Th2) responses during infection by *Leishmania* sp.^10−13^

*L. major*, the focus species of this study, has an array of mechanisms that contribute to the establishment of the disease. For example, *L. major* can block the activity of protein kinase C (PKC), thereby interrupting the formation of the parasitophorous vacuoles to favor its persistence in the intracellular environment.^14,15^ Furthermore, *L. major* favors the peroxisome proliferator-activated receptor γ (PPAR-γ) signaling pathway, resulting in the suppression of inflammation and inhibition of parasite death.^16^ The PPAR-γ pathway is also known to positively regulate the pathway of cyclooxygenase-2 (COX-2), an enzyme that produces lipid mediators such as prostaglandin E_2_ (PGE_2_). Some authors have associated the increased production of PGE_2_ with enhanced parasite survival mechanisms, while high levels of Leukotriene B4 (LTB_4_) are linked to better control of infection in host cells.^17−19^ Other studies have demonstrated that constituents of *L. major*, such as LPG and gp63, can interfere with the activation of immune system responses.^20−22^

Lipophosphoglycan (LPG) is the most abundant glycoconjugate in *Leishmania,* and several studies have shown that intra- and interspecies polymorphisms are important for macrophage/neutrophil activation. For instance, LPGs from different dermotropic strains of *Leishmania amazonensis* and *Leishmania braziliensis* can trigger various immune responses in murine macrophages via TLR4/TLR2.^23,24^ This pattern is also observed in dermotropic/viscerotropic *Leishmania infantum*.^25^ Regarding neutrophils, *L. amazonensis* LPG induces the formation of neutrophil extracellular traps (NETs) and the production of LTB_4_.^18,26^ As expected, LPG-deficient *L. infantum* is more vulnerable to being killed by neutrophils/macrophages.^27,28^ In the case of dermotropic *L. major*, TLR2 is the main receptor for its LPG.^30,31^

LPGs from *L. major* strains (FV1 and LV39) have been fully characterized.^29−31^ The LPG of FV1 strains has short side chains bearing galactose, which is often capped with arabinose residues, while the LPGs of LV39 strains have long galactose side chains.^29,31^ However, data addressing the role of intraspecific polymorphisms in *L. major* LPG and their functional properties are scarce. Here, we tested the proinflammatory response of polymorphic LPGs from two *L. major* strains (FV1 and LV39) during their interactions with macrophages.

## Results

### LPG from the *L. major* Strain LV39 Induces Nitric Oxide and PGE_2_ Production via iNOS and COX-2

The FV1 and LV39 Leishmania strains present different amounts and compositions of side chains in their LPG molecules. To evaluate whether these polymorphisms activated macrophages differently, bone marrow-derived macrophages (BMDM) primed with IFN-γ were stimulated with LPGs from LV39 and FV1 strains, and the production of NO and PGE2 was assessed.

**Figure 1:**
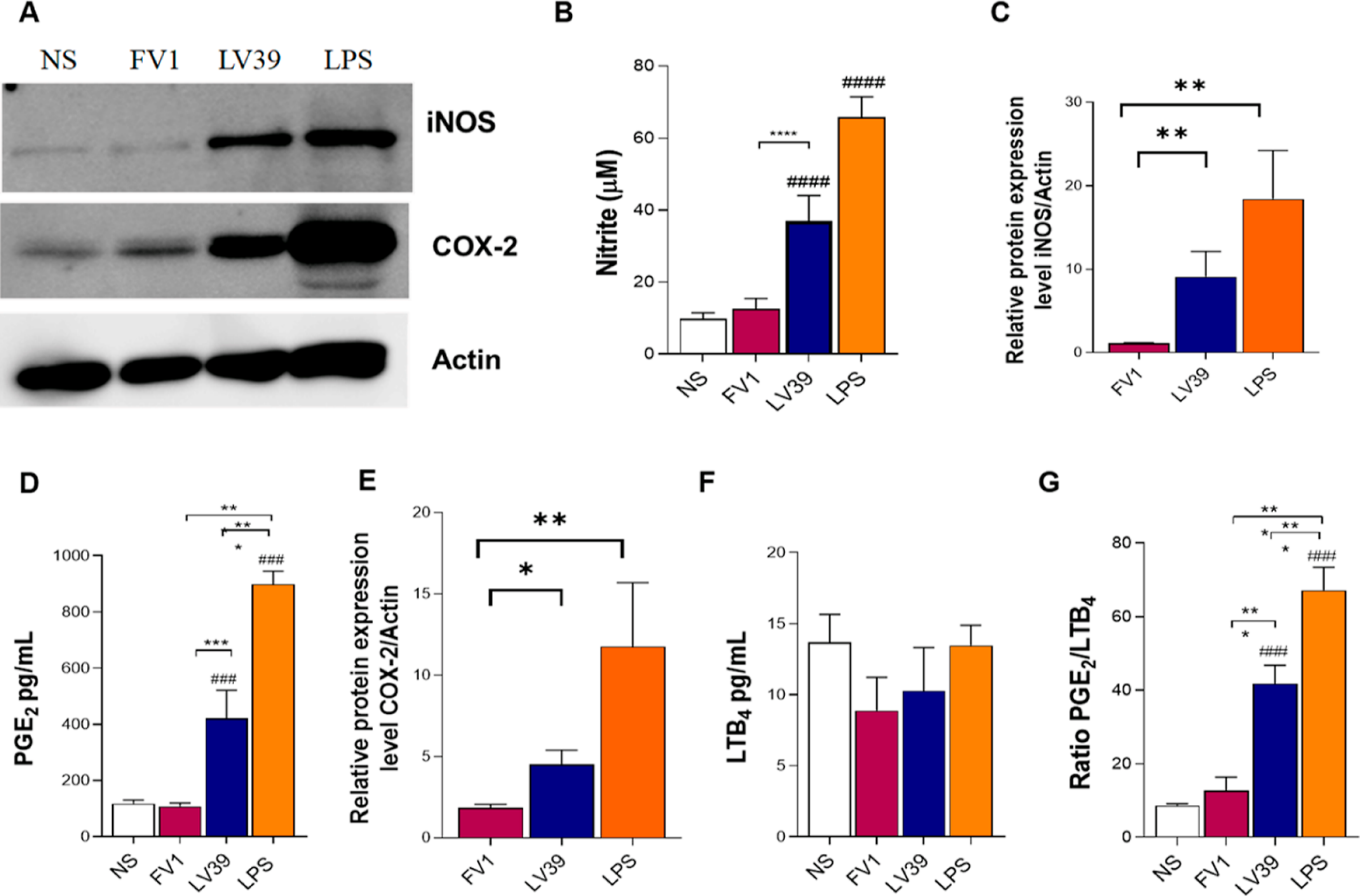
Purified LPG from *L. major* strain LV39 induces higher nitric oxide and PGE2 production via iNOS and COX-2. BMDM primed with IFN-γ (100 ng/mL) were stimulated for 24 h with 10 μg/mL of LPG purified from *L. major* FV1 or LV39. LPS (300 ng/mL) was used as a positive control. (A) Inos and COX-2 Western blot analysis from cell lysate 24 h after LPG stimulation. Actin was used as a loading control. (B) Nitric oxide (NO) production was measured in culture supernatants by the Griess reaction. (D) PGE_2_ and (F) LTB_4_ were dosed in culture supernatants by ELISA. (G) PGE_2_/LTB_4_ ratio. Bars represent means ± SD of quadruplicates. Representative densitometry ratios of iNOS bands (C) and COX-2 bands (E) by actin bands (C,E) were obtained by ImageJ/Fiji. The statistical analysis was performed using the ANOVA test followed by the Newman–Keuls post-test using multiple comparisons between the experimental groups (****p < 0.0001) and (####*p* < 0.0001) compared to the control group. NS: nonstimulated; FV1 and LV39: *L. major* LPGs. LPS: lipopolysaccharide.

Macrophages stimulated with the purified LPG from the LV39 strain produced higher levels of NO compared to those stimulated with FV1 LPG (Figure 1B). Consistent with these observations, the level of iNOS expression induced by LV39 LPG was also higher (Figure 1A,C). Additionally, LV39 LPG induced greater production of PGE_2_ compared to FV1 LPG (Figure 1D), with no effect on LTB_4_ production (Figure 1D). However, the PGE_2_/LTB_4_ ratio was increased for LV39 LPG, confirming that this glycoconjugate preferentially induces PGE_2_ production in macrophages rather than LTB_4_ (Figure 1G). Similar to PGE_2_, the level of induction of COX-2 in macrophages stimulated with LV39 LPG was higher than that in those stimulated with FV1 LPG (Figure 1A,E). These results suggest that LPG from LV39 promotes more robust macrophage activation when compared to the LPG from the FV1 strain.

### Purified LPG from FV1 and LV39 Induces Differential ERK and JNK Pathway Activation

Previous work has demonstrated the importance of the ERK and JNK (MAPK) pathways in macrophage activation during Leishmania infection in response to LPG recognition.^32−34^ Here, we evaluated the involvement of these pathways in macrophage activation by LPGs isolated from the FV1 and LV39 strains.

**Figure 2:**
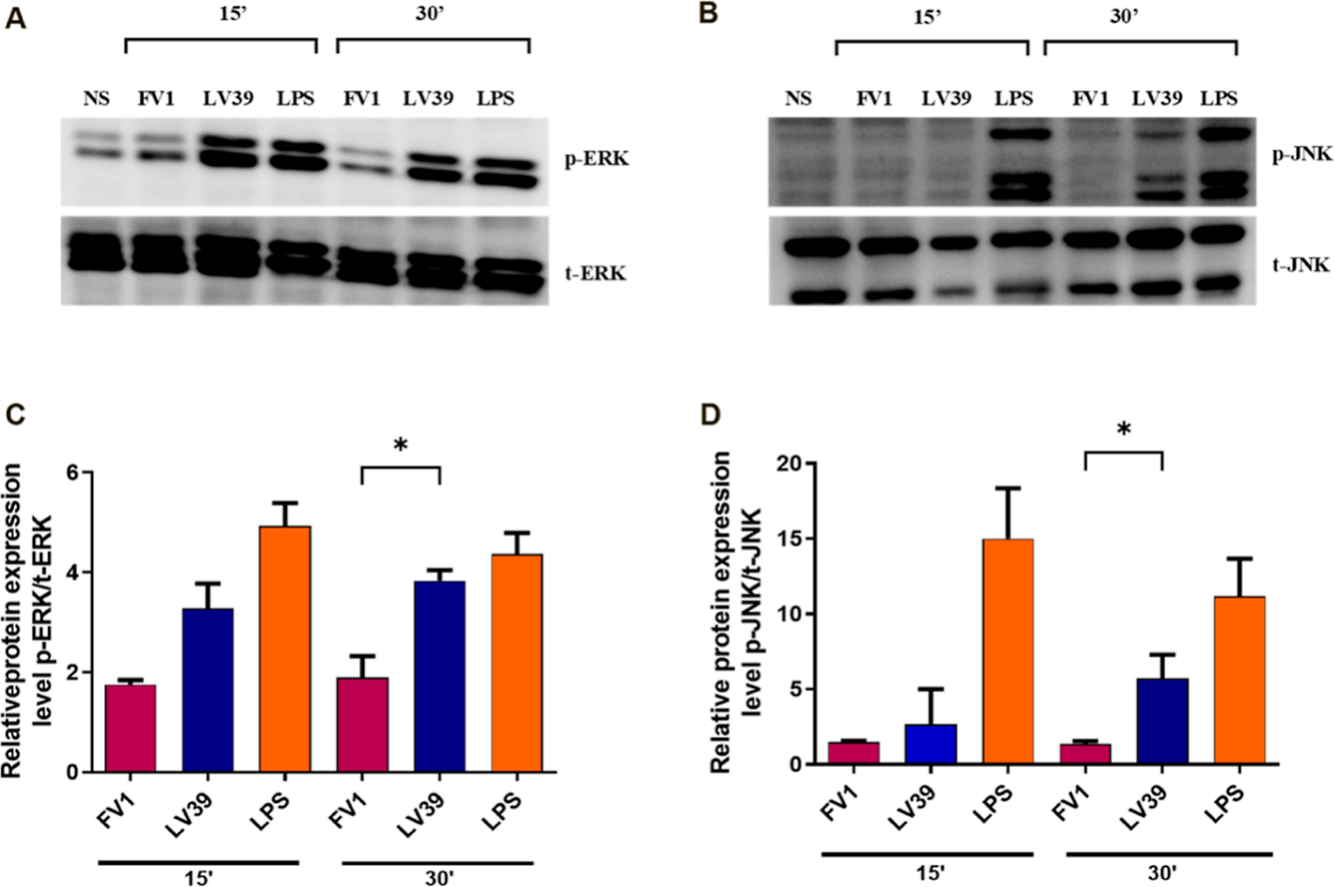
Signaling pathways triggered by purified LPG from *L. major*. BMDM were stimulated with 10 μg/mL of purified LPG from the FV1 or LV39 strains for 5, 15, 30, and 60 min. LPS (300 ng/mL) was used as a positive control. The bands indicate phosphorylation of ERK1/2 (A) and JNK (B) in cell lysates evaluated by Western blotting. Representative densitometry ratios of *p*-ERK (C) and *p*-JNK (D) by total-ERK expression were obtained by ImageJ/Fiji. The statistical analysis was performed using the ANOVA test followed by the Newman–Keuls post-test using multiple comparisons between the experimental groups (*p < 0.05). NS: nonstimulated; FV1 and LV39: *L. major* LPGs. LPS: lipopolysaccharide.

To define the role of LPGs in ERK and JNK signaling pathways, macrophages were exposed to these glycoconjugates, and pathway activation was evaluated by Western blot. FV1 LPG induced lower JNK phosphorylation compared to LV39 LPG. Between 15 and 30 min, an increase in JNK activation was detected only with LV39 LPG (Figure 2B,D). Both FV1 and LV39 LPGs induced a gradual activation of ERK1/2, but this activation started earlier for the FV1 LPG (Figure 2A,C). Total ERK and JNK were used as the normalizers (Figure 2A,C, bottom panel). These data suggest that both LPGs are capable of activating the MAPK pathways in macrophages but with different patterns.

### LPG from *L. major* LV39 Induces an Increase in TNF-α Production

**Figure 3:**
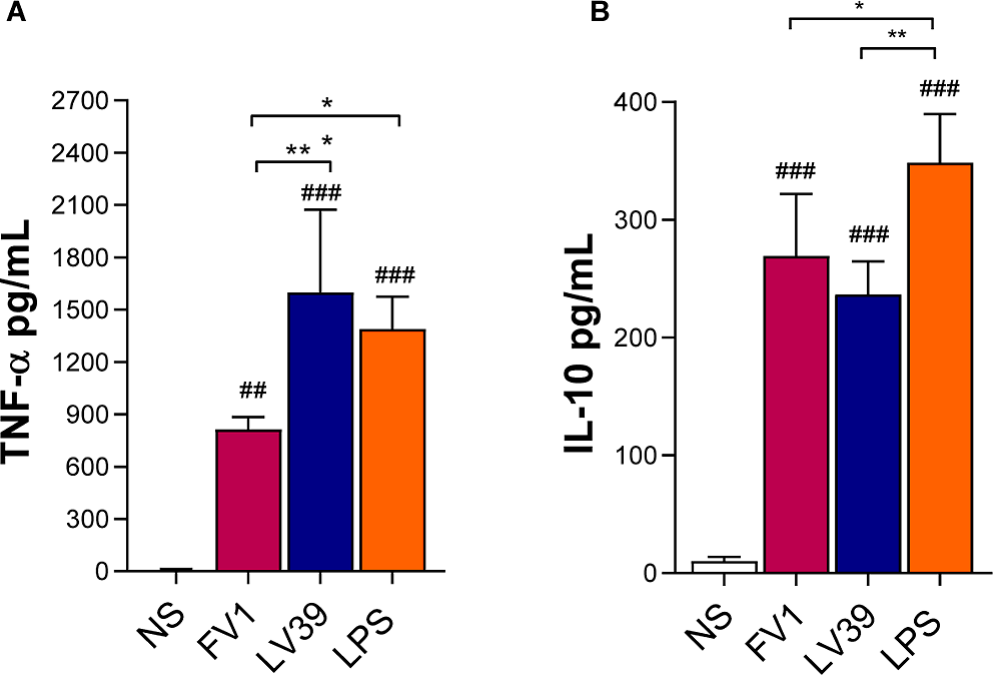
Impact of purified *L. major* LPG cytokine production. BMDM primed with IFN-γ (100 ng/mL) were stimulated for 24 h with 10 μg/mL of LPG purified from *L. major* FV1 or LV39. LPS (300 ng/mL) was used as a positive control. The production of (A) TNF-α and (B) IL-10 was evaluated from culture supernatants. Bars represent means ± SD performed in quadruplicates. The analysis was performed using the ANOVA test followed by the Newman–Keuls post-test using multiple comparisons between the experimental groups (*p < 0.05; **p < 0.01) and (##*p* < 0.01; ###*p* < 0.001) when compared to the NS control group). Ns: nonstimulated; FV1 and LV39: *L. major* LPGs. LPS: lipopolysaccharide.

Since the recognition of purified LPG by macrophages through pattern recognition receptors (PRRs) leads to the production of inflammatory mediators and cytokines, we assessed whether the activation of ERK1/2 and JNK signaling pathways could be involved in cytokine production and thus impact inflammation. The levels of TNF-α and IL-10 were measured in the supernatant of cell cultures stimulated by LPG. TNF-α production was higher in macrophages stimulated with LV39 LPG (Figure 3A). No difference in IL-10 production was observed after stimulation with both LPG, and they were lower than that of the positive control LPS (Figure 3B). Both of the LPGs tested were able to differentially induce TNF-α and IL-10.

### ERK, COX-2, PKC, and PPAR-γ Are Involved in NO-Production and Cytokine Production Induced by LV39 LPG

LV39 LPG, described as the most virulent strain,^35,36^ was a better inducer of several mediators in macrophages compared to FV1. Therefore, the subsequent experiments focused on confirming and expanding the characterization of the pathways involved with LV39 LPG-induced macrophage activation. To this end, we used pharmacological inhibition/activation of signaling pathways that were previously described to be involved in the recognition of LPGs from different species.

**Figure 4:**
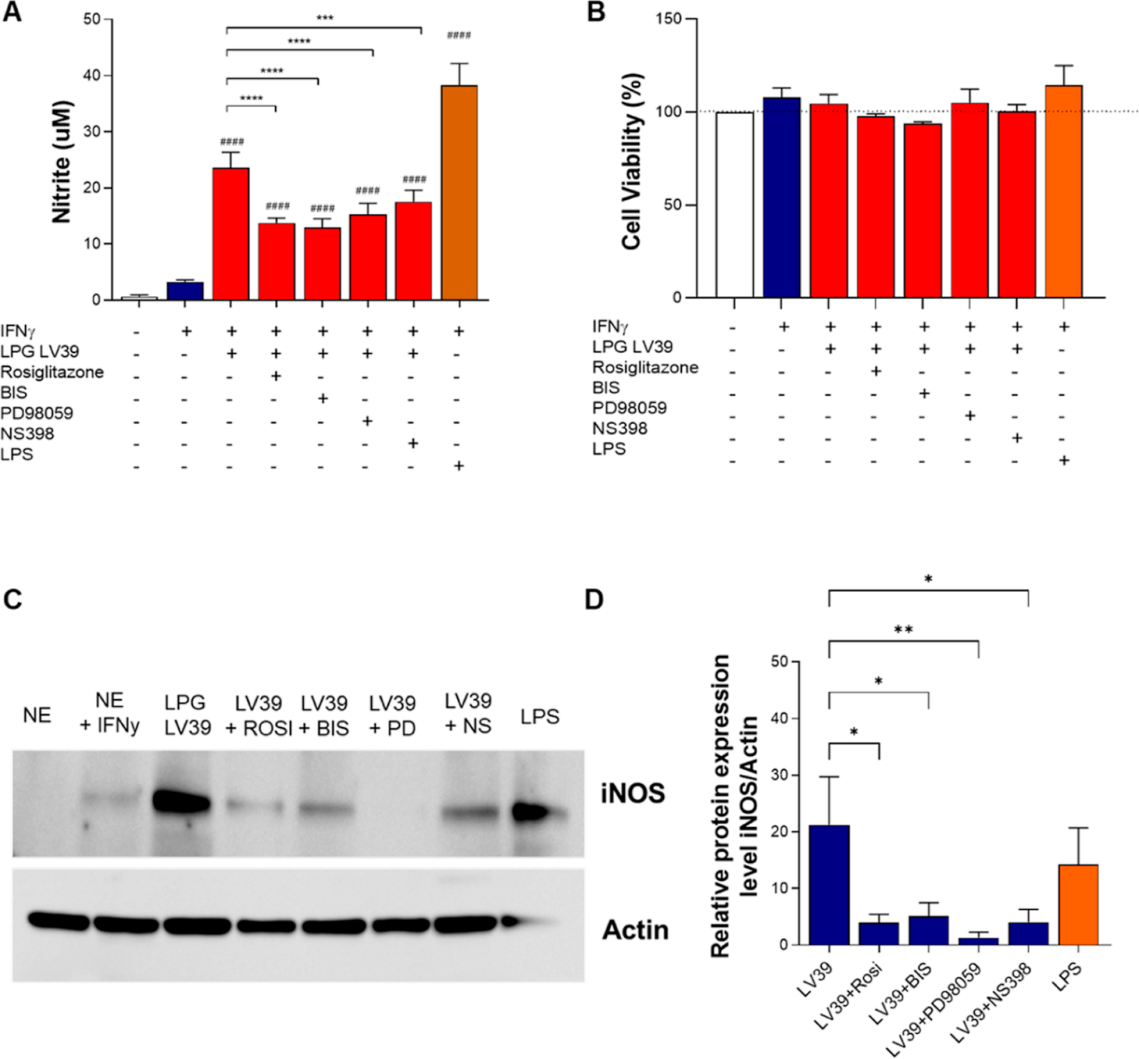
Inhibition of ERK, COX-2, and PKC pathways and induction of PPARy negatively impact the capacity of nitric oxide production by *L. major* LPG LV39. BMDM were primed with IFN-γ (100 ng/mL) for 24 h. Afterward, they were pretreated for 1 h with 10 μM rosiglitazone (PPAR-γ agonist), 20 μM BIS (PKC inhibitor), 50 μM PD98059 (ERK1/2 inhibitor), or 1 μM NS398 (COX-2 inhibitor). Then, the stimulus with purified LPG LV39 was added for 24 h. (A) Nitric oxide (NO) production was measured in culture supernatants by the Griess reaction. (B) Cell viability was assessed by Alamar Blue. (C) Bands were obtained by Western blotting from culture lysates. (D) Representative densitometry quantification iNOS and actin expression were obtained by ImageJ/Fiji. Bars represent means ± SD of experiments performed in quadruplicates. The analysis was performed by an ANOVA test followed by the Newman–Keuls post-test used for multiple comparisons between experimental groups (***p < 0.001; ****p < 0.0001, and ####*p* < 0, 0001 when compared to the NS control group). NS: nonstimulated; FV1 and LV39: *L. major* LPGs. LPS: lipopolysaccharide.

Macrophages were pretreated for 1 h with rosiglitazone (PPAR-γ agonist), BIS (PKC inhibitor), PD98059 (ERK1/2 inhibitor), and NS-398 (COX-2 inhibitor) prior to stimulation with LPG. The supernatant was collected for cytokine and NO measurement, and cell lysates were assessed for iNOS production. Pharmacological intervention decreased NO production without affecting macrophage viability (Figure 4A,B). Consistent with these observations, a reduction in the level of iNOS expression was detected in the cell lysates (Figure 4C,D).

**Figure 5:**
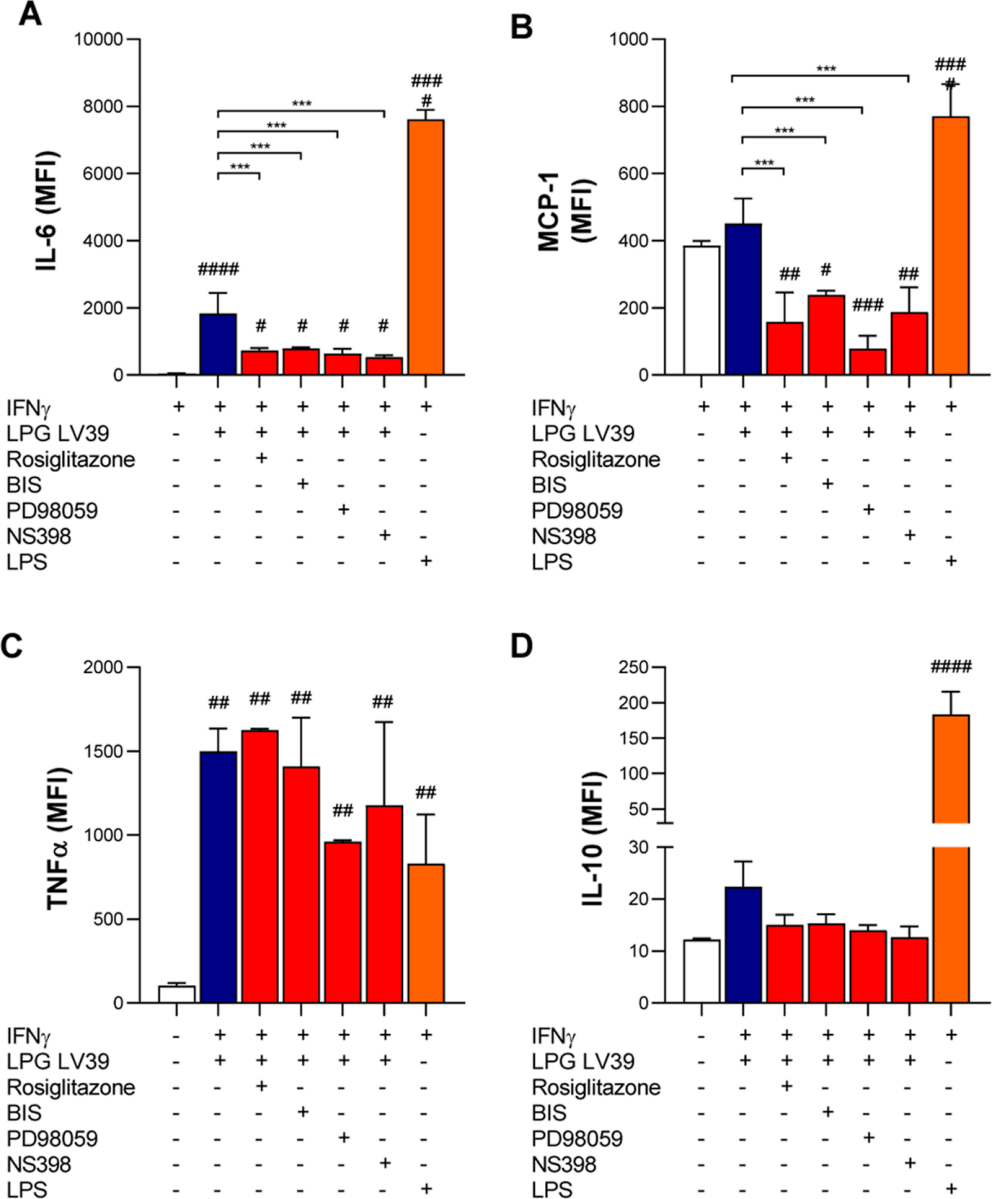
Inhibition of ERK, COX-2, and PKC pathways and induction of PPAR-γ alter the ability to produce IL-6 and MCP-1 by macrophages stimulated with *L. major* LPG LV39. BMDM primed with IFN-γ (100 ng/mL) were pretreated with rosiglitazone (PPAR-γ agonist), BIS (PKC inhibitor), PD98059 (ERK1/2 inhibitor), and NS398 (COX-2 inhibitor) for 1 h. Then, cells were stimulated for 24 h with 10 μg/mL of LPG purified from *L. major* FV1 or LV39. LPS (300 ng/mL) was used as a positive control. (A) IL-6, (B) MCP-1, TNF-α (C), and IL-10 (D) dosage was performed with culture supernatants by Luminex. Bars represent means ± SD of experiments performed in quadruplicates. The analysis was performed using the ANOVA test followed by the Newman–Keuls post-test used for multiple comparisons between the experimental groups (***p < 0.001) and (##*p* < 0.01; ###*p* < 0.001 ####*p* < 0.0001) compared to the NS control group). NS: nonstimulated; FV1 and LV39: *L. major* LPGs. LPS: lipopolysaccharide.

Similar to NO, IL-6 and MCP-1 production was partially affected by all inhibitors (Figure 5A,B), with no effect on TNF-α and IL-10 (Figure 5C,D). Together, these results demonstrate that LPG from LV39 elicits a PKC-ERK-JNK pathway to produce NO and inflammatory cytokines. The LPG-induced macrophage activation is modulated by PPAR-γ.

## Discussion

Specific constituents on the surface of *Leishmania*, such as LPG, contribute to the parasite’s virulence and pathogenicity by triggering evasion mechanisms against the host’s cellular responses. Variations and functions of LPG have been reported in the last two decades in both Old and New World species of *Leishmania*.^23−25,37^ Here, we used two LPGs purified from two *L. major* strains (FV1 and LV39) that differ in size and galactosylation levels to evaluate their effect on inflammatory activation in macrophages.

Purified LPGs from both *L. major* strains induced the differential activation of cellular immune responses in murine macrophages primed with IFN-γ.^38,39^ We observed that NO production was more pronounced in macrophages stimulated with LV39 LPG. Additionally, there was a higher expression of the iNOS enzyme in macrophages stimulated with LV39 compared with those stimulated with FV1. Therefore, we suggest that LPG from the LV39 strain induces NO production via iNOS in macrophages. Since previous studies have shown that the LPG from the LV39 strain has larger polygalactosylated side chains than the LPG from the FV1 strain,^31^ our results may suggest that these structural modifications result in the differential activation of macrophages and the promotion of distinct inflammatory responses.

LPG can activate innate response receptors, leading to the production of reactive oxygen and nitrogen species, as well as inflammatory mediators.^38^ PGE_2_ and LTB_4_ are lipid mediators with crucial roles in innate immunity during *Leishmania* spp. infection.^39−41^ To evaluate the inflammatory potential of these LPGs further, we measured the production of lipid mediators, such as PGE_2_ and LTB_4_, after stimulating BMDM with purified LPGs from *L. major*. Our results showed that LPG from the *L. major* LV39 strain led to an increased PGE_2_/LTB_4_ ratio compared to LPG from the FV1 strain. In agreement with the literature, this elevated production of PGE_2_ induced by LV39 LPG may provide a benefit to the parasite, enhancing its virulence compared to that of the FV1 strain.

The existence of intra- and interspecific structural variations in LPG molecules from *Leishmania* spp., whether from the New or Old World, results in the differential activation of macrophages, which affects NO production, cytokine levels, and MAPK pathway activation.^42,43^ Our findings align with previous work from our group, which demonstrated that LPG from *L. infantum* is associated with increased PGE_2_ production through COX-2 induction.^34^ Furthermore, LPGs from different strains of *L. infantum* have been shown to induce PGE_2_ and NO production differentially in macrophages.^27,34^ Thus, the increase in PGE_2_ production observed with LV39 LPG is likely a result of enhanced COX-2 expression.

After determining that LPGs from different *L. major* strains induced varying levels of NO and PGE_2_ production, we proceeded to investigate the signaling pathways that they triggered. We observed distinct kinetics in the ERK and JNK signaling in LPG-stimulated macrophages. While both LPGs progressively activated JNK, LV39 LPG elicited a more intense response. Early ERK phosphorylation was observed in macrophages stimulated with FV1 LPG, but LV39 LPG induced more pronounced ERK phosphorylation after longer stimulation periods. These findings indicate that LPG purified from *L. major* strains drives distinct responses, with LV39 LPG promoting a more robust and complex signaling cascade in macrophages compared to FV1 LPG.

To understand whether the purified *L. major* LPGs were involved in additional mediator production, we measured cytokines in the culture supernatant of stimulated cells. Both FV1 and LV39 LPGs induced the production of TNF-α and IL-10. However, LV39 LPG stimulated a higher production of TNF-α in murine macrophages, suggesting a role for *L. major* LPG in driving inflammatory responses during parasite infection. These results support the idea that LPG from the LV39 strain leads to intense macrophage activation, resulting in a more pro-inflammatory profile with increased production of cytokines, such as TNF-α. Intraspecific polymorphisms in *L. major* LPG are sufficient to promote different levels of cell activation and result in varying macrophage responses.

Additionally, we investigated whether other signaling pathways converged with the NO-production pathway and cytokine production induced by LV39 LPG using specific inhibitors. Our results demonstrated that activating the PPAR-γ pathway (with rosiglitazone) and inhibiting the PKC (with BIS), ERK1/2 (with PD98059), and COX-2 (with NS398) pathways impaired the production of IL-6 and MCP-1 induced by LV39 LPG. However, TNF-α and IL-10 production remained unchanged in the presence of these inhibitors or agonists. Likewise, we observed a decrease in the level of NO production and iNOS expression when these pathways were inhibited or stimulated.

There is evidence in the literature that strains related to LV39 are more resistant to infection and induce more severe lesions than strains related to FV1.^35,36^ In vitro assays demonstrate that the *L. major* strain LV39 is more resistant to being killed than the FV1 strain (Friedlin clone strain) in IFN-*g*-activated peritoneal macrophages.^35^ Recently, a study suggested that initial differences in macrophage activation between two *L. major* strains, one virulent (5-ASKH, the same geographic origin as LV39) and one avirulent (FV9, Friedlin FV1), contributed to increased inflammation and tissue damage.^36^ This differential macrophage activation was dependent on TLR2, a PRR previously described to recognize LPG.^36,42^

In conclusion, as a part of a broader study of the functional properties of *Leishmania* glycoconjugates, our results confirm that LPG polymorphisms differentially affect macrophage activation and may influence the virulence and disease outcomes of dermotropic and viscerotropic *Leishmania* species.

## Methods

### Ethics Statement

For all experiments, male BALB/c mice aged between 6 and 8 weeks were used, in accordance with the recommendations of the Brazilian Council for the Control of Animal Experimentation (CONCEA). The study was approved by the Internal Committee for Ethics in Animal Experimentation of the Gonçalo Moniz Institute of the Oswaldo Cruz Foundation, Salvador, Bahia (CEUA-IGM-FIOCRUZ), with the protocol number 021/2015.

### Parasites, Cell Culture, and LPG Purification

LPGs were purified from two World Health Organization reference strains of *L. major,* namely, MHOM/IL/80/Friedlin (FV1) and MRHO/SU/59/P (LV39). Parasites were grown in M199 supplemented with 10% FBS. In summary, parasite pellets were subjected to organic extraction, and LPGs were purified by hydrophobic interaction in a phenyl-sepharose column. LPGs were quantified using the phenol-sulfuric method.^44^

#### Obtaining Bone Marrow Macrophages

BMDM were obtained from BALB/C mice by washing the inside of the femur and tibia with RPMI 1640 (LGC) supplemented with 20% inactivated bovine serum (SBI) and 30% L929 cell supernatant (LCCM), 1% l-glutamine, and 1% penicillin, as previously reported.^34^ The cells were homogenized and distributed in Petri dishes and kept in an incubator at 37 °C and 5% CO_2_ for 7 days to achieve their differentiation. After 1 week, the cells were harvested on ice, counted, and plated in a 96-well plate at a density of 1 × 10^5^ cells per well for experiments.

#### Cell Treatment and Cytokines, PGE_2_, and LTB_4_ Measurement

Differentiated BMDM were prestimulated with 100 ng/mL of IFN-γ for 24 h. After this period, they were incubated with 10 μg/mL of LPG purified from *L. major* of FV1 or LV39 for an additional 24 h. We used 300 ng/mL of bacterial LPS as a positive control in all experiments. After the incubation period, the supernatants were removed for cytokine/chemokine measurement using a Milliplex mouse cytokine/chemokine (MCYTOMAG-Merck), ELISA TNF-α (Mouse TNF-alpha DuoSet ELISA catalog #DY410, R&D Systems), and ELISA IL-10 (Mouse IL-10 DuoSet ELISA catalog #DY417, R&D Systems) according to the manufacturer’s instructions. The dosage of PGE_2_ (Prostaglandin E2 ELISA Kit-Cayman Chemical catalog #514010) and LTB_4_ (ELISA Kit-Cayman Chemical catalog #502390) was also performed on the supernatant of stimulated cells, as described above, using the ELISA technique, as recommended by the manufacturer of the dosage kit. The reading was performed with the SpectraMax M Series Multi-Mode Microplate Readers at 450 nm.

#### Determination of Nitric Oxide (NO)

The determination of NO was performed on the supernatant of stimulated cells, as described above, using an indirect detection method called the Griess reaction. This method can detect nitrite (NO^2–^) in liquid, biological, and experimental matrices. For the determination of the nitrite (NO^2–^) concentration, 50 μL of the culture supernatant was used, with the addition of 50 μL of the Griess Reagent System (1:1 of sulfanilamide and *N*-1-naphthylethylenediamine dihydrochloride). The preparation of a standard curve was necessary to generate an accurate quantification of NO^2–^. The reading was performed with the SpectraMax M Series multi-mode microplate readers at 540 nm.

### Western Blot

After protein extraction with RIPA buffer, the extracts were quantified using BCA (Pierce BCA protein assay kits catalog #: A55864), and 50 ng of total protein was subjected to sodium dodecyl sulfate-polyacrylamide gel electrophoresis and 10% polyacrylamide gel electrophoresis and was then transferred to a nitrocellulose membrane with a Turbo-Blotting BIORAD. After blocking with 5% milk or BSA (for phosphorylated proteins) for 1 h, the membranes were incubated for an additional 1 h with the respective primary antibodies diluted in TBS-T 0.05%. For this, we used the following: antiphosphorylated-ERK-1/2 antibodies (dilution 1:1000 Phospho-p44/42 MAPK (Erk1/2) (Thr202/Tyr204) (D13.14.4E) XP Rabbit mAb catalog #4370 Cell Signaling Technology), antiphosphorylated-JNK (dilution 1:1000 Phospho-SAPK/JNK (Thr183/Tyr185) (98F2) Rabbit mAb catalog # 4671 Cell Signaling Technology), antitotal-ERK (dilution 1:1000 p44/42 MAPK (Erk1/2) (137F5) Rabbit mAb catalog #4695 Cell Signaling Technology), actin (dilution 1:1000 Monoclonal Anti-Actin antibody produced in mouse catalog #4700 Sigma-Aldrich-Merck), anti-Inos (dilution 1:1000 iNOS Polyclonal antibody catalog #160862 Cayman chemical), and anti-cox-2 (dilution 1:1000 COX-2 (mouse) Polyclonal antibody (aa 570–598) catalog #160106 Cayman chemical). As a secondary antibody, we used anti-Mouse IgG HRP-conjugated (dilution 1:3000 KPL Peroxidase-Labeled Affinity Purified Antibody to Mouse IgG (H + L) [Goat] catalog no. 5450-0011 KPL-SeraCare) and antirabbit IgG HRP-conjugated (dilution 1:3000 KPL Peroxidase-Labeled Affinity Purified Antibody to Mouse IgG (H + L) [Goat] catalog #5450–0010 KPL-SeraCare). Among all steps, three washes, each lasting 5 min, were carried out under agitation with TBS-T containing 0.05% Tween 20. Pierce ECL Western Blotting Substrate Catalog #. 32106 rRNA was used to detect protein expression. Bands were acquired on an ImageQuant LAS 4010 GE Healthcare-Cytiva.

#### Macrophage Toxicity Assay

Cells were prestimulated with IFN-γ (100 ng/mL) for 24 h prior to treatment with 10 μM rosiglitazone (PPAR-γ agonist), Cayman Chemical catalog #7174, 20 μM BIS (PKC inhibitor), Bisindolylmaleimide II Sigma-Aldrich-Merck catalog #B3056, 50 μM PD98059 (ERK1/2 inhibitor), Tocris catalog #1213 or 1 μM NS398 (COX-2 inhibitor), and Tocris catalog #0942 for 1 h. Afterward, cells were exposed to LPG LV39 (10 ng/mL) and LPS (300 ng/mL-control). Cells were then reincubated for 4 h with supplemented RPMI medium containing 10% Alamar Blue. Unstimulated cells were incubated with RPMI medium only. The reading was performed using the SpectraMax M Series Multi-Mode Microplate Readers at 570 and 600 nm.

### Statistical Analyses

The Shapiro–Wilk normality test was performed (*n* < 30), and the p-value >0.05 demonstrated that the data did not deviate from the Gaussian distribution (parametric distribution). Thus, the ANOVA test was performed to test the equality of means between independent groups, with the post-Newman–Keuls test used for multiple comparisons between experimental groups. All experiments were performed at least three times and in quadruplicates, with one animal for each experiment. The data obtained were represented by mean ± standard deviation and representative experiments. All analyses were performed using GraphPad Prism 8.0 software (GraphPad Software, San Diego, CA, USA) Windows version, where *p* < 0.05 was considered significant.

## Supporting Information Available

Western blot analysis for iNOS, COX-2, and Actin; Western blot analysis for *p*-ERK, *p*-JNK, Total ERK, and total JNK; and Western blot analysis for iNOS and actin across different experimental treatments (PDF)
