# Supplementary material for: Activation Pathways of Murine Macrophages by Lipophosphoglycan from Strains of Leishmania major (FV1 and LV39)
Source: ACS Infect Dis. 2024 Sep 23;10(10):3544–52. doi: 10.1021/acsinfecdis.4c00295 (PMC11474971; doi:10.1021/acsinfecdis.4c00295)
Supplement: Supplementary file 1 — id4c00295_si_001.pdf [file id4c00295_si_001.pdf]

ACTIVATION PATHWAYS OF MURINE MACROPHAGES BY LIPOPHOSPHOGLYCAN FROM STRAINS OF *LEISHMANIA MAJOR* (FV1 AND LV39)

Vanessa Mançur Santos,[1,2] Astrid Madeleine Calero Goicochea,[1,2] Antônio José Soares Neto,[3] Flávio Henrique Jesus Santos,[1,2] Jéssica Lobo da Silva,[1,2] Theo Araújo-Santos,[3] Leonardo Paiva Farias,[1,2] Claudia Ida Brodskyn,[1,2] Valéria M. Borges,[1,2] Rodrigo Pedro Soares,[*,4,†] and Jonilson Berlink Lima[*,3,†]

[1] Instituto Gonçalo Moniz, Fundação Oswaldo Cruz (FIOCRUZ), Salvador, BA 40296-710 - Brasil

[2] Faculdade de Medicina, Universidade Federal da Bahia (UFBA), Salvador, BA 40.026-010 - Brasil

[3] Núcleo de Agentes Infecciosos e Vetores (NAIVE), Universidade Federal do Oeste da Bahia (UFOB), Barreiras, BA 47808-021 - Brasil

[4] Instituto René Rachou, Fundação Oswaldo Cruz (FIOCRUZ), Belo Horizonte, MG 30.190-009- Brasil

[†]These senior authors contributed equally to the work.

*Corresponding authors:

Dr. Jonilson Berlink Lima, e-mail: jonilson.lima@ufob.edu.br

Rodrigo Pedro Pinto Soares, e-mail: rodrigo.pedro@fiocruz.br

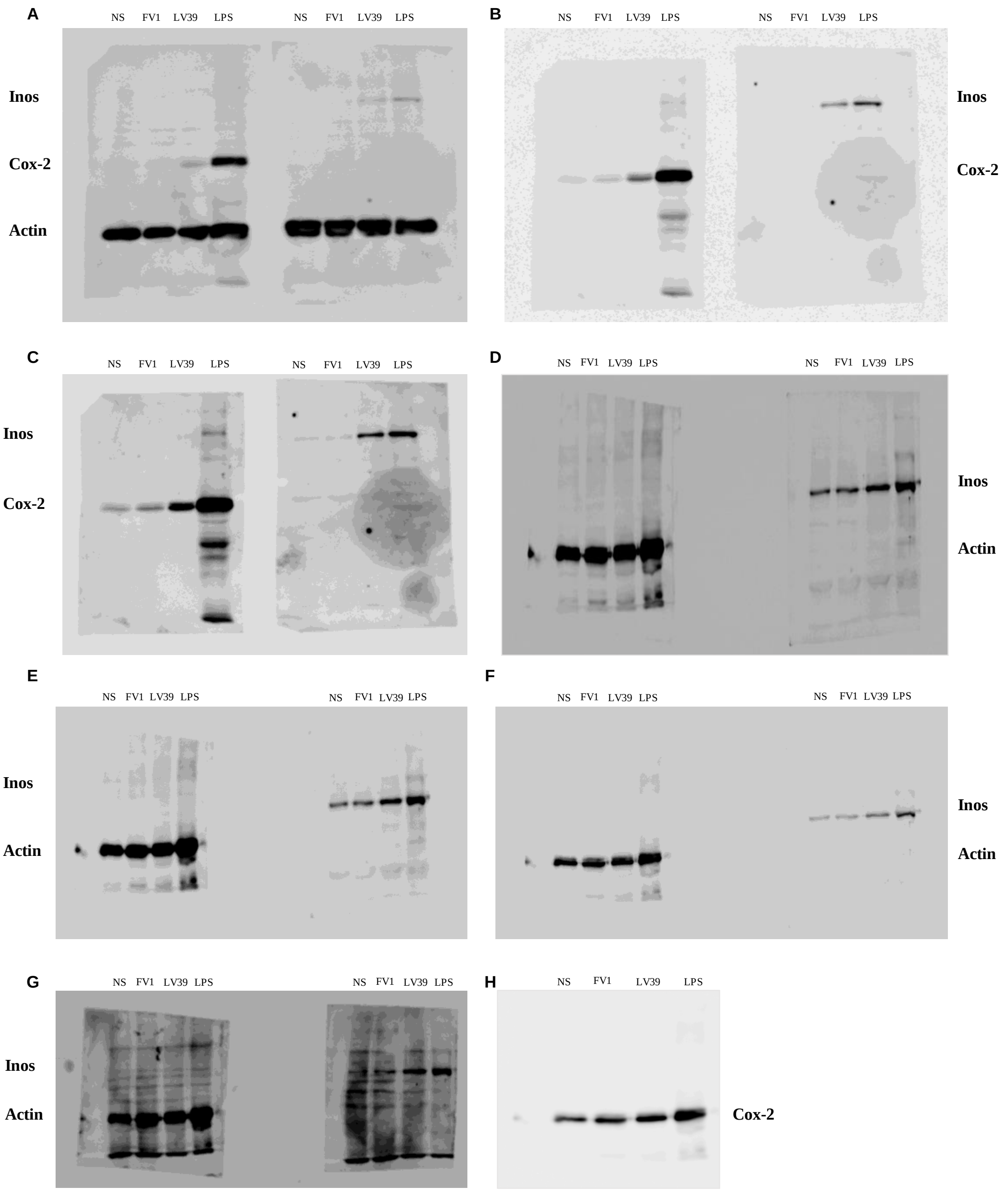

Figure S1 Supplementary: Western Blot Analysis for iNOS, COX-2, and Actin

(A-H) Western blot images showing the expression levels of iNOS, COX-2, and Actin. Each panel (A-H) corresponds to different experimental conditions or replicates. Actin is included as a loading control. The full blots are provided to verify the correct size of the bands and ensure the quality of the Western blots.

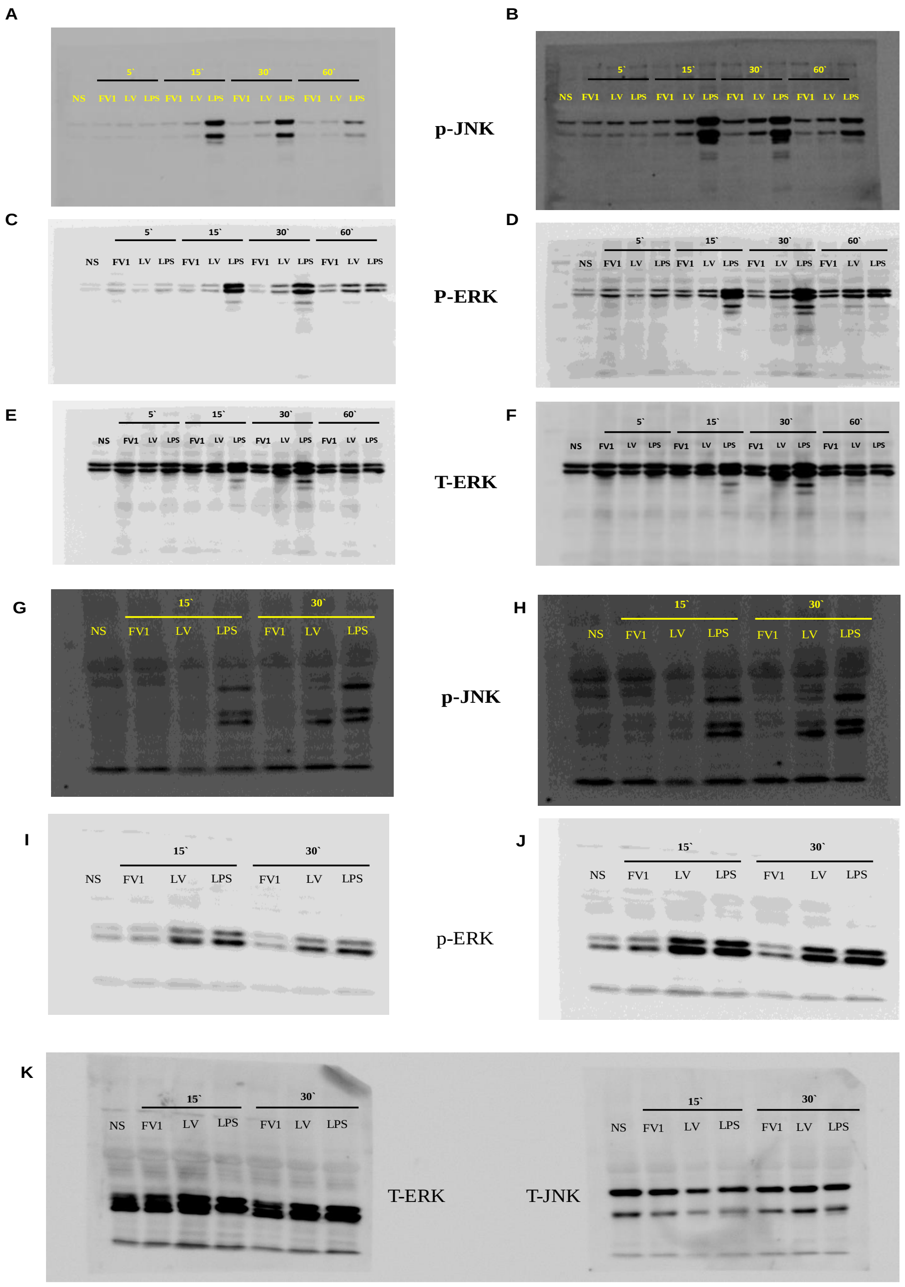

Figure S2 Supplementary: Western Blot Analysis for p-ERK, p-JNK, Total ERK, and Total JNK

(A-K) Western blot images showing the expression levels of phosphorylated ERK (p-ERK), phosphorylated JNK (p-JNK), total ERK, and total JNK under various experimental conditions. Each panel (A-K) represents different experimental conditions or replicates, providing a comprehensive overview of the activation states and total levels of ERK and JNK. The full blots are provided to verify the correct size of the bands and ensure the quality of the Western blots.

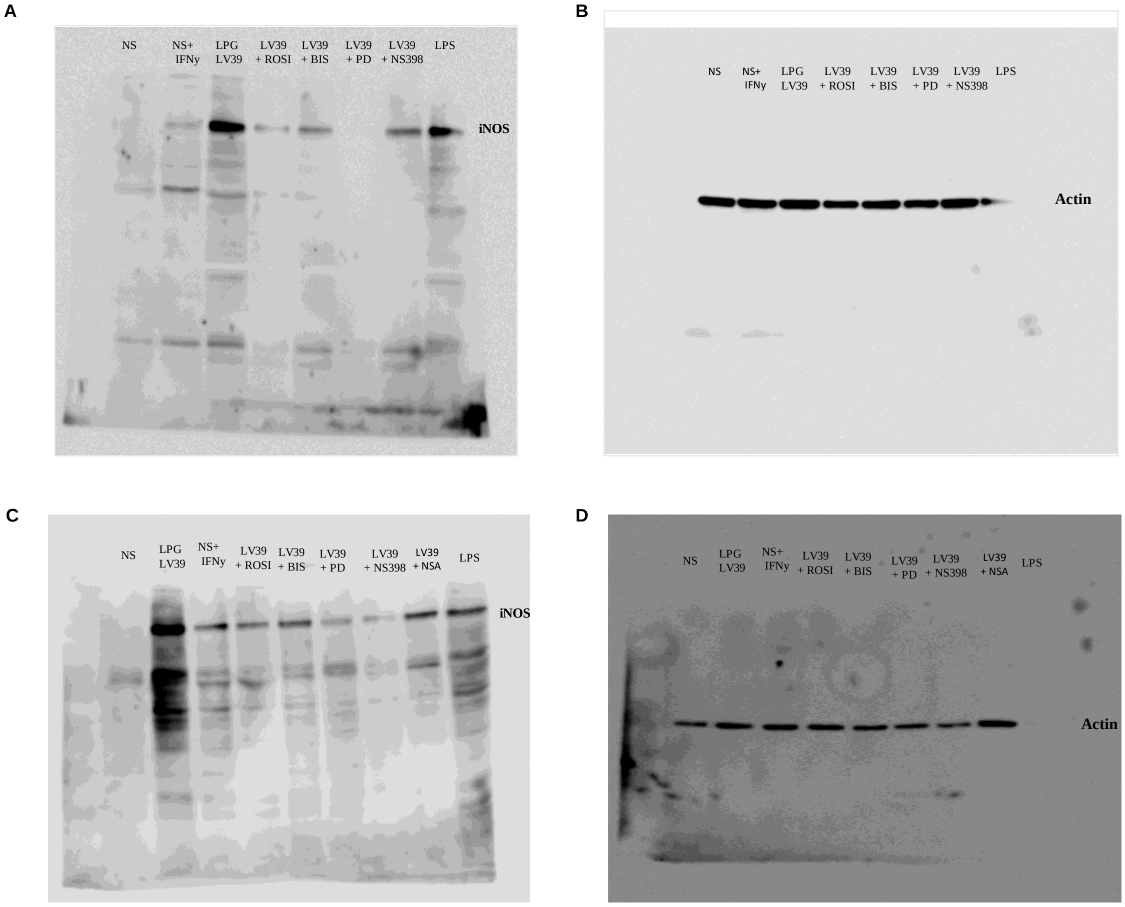

Figure S3 Supplementary: Western Blot Analysis for iNOS and Actin Across Different Experimental Treatments

(A-D) Western blot images showing the expression levels of iNOS and Actin across various experimental treatments. The groups include treatments with interferon gamma (IFN-γ), Rosiglitazone (PPAR-γ inhibitor), Bisindolylmaleimide II (PKC inhibitor), PD98059 (ERK inhibitor), and NS398 (COX-2 inhibitor). Actin is included as a loading control to ensure equal protein loading across the samples. The full blots are provided to verify the correct size of the bands and ensure the quality of the Western blots.
